# Supplementary material for: Charge carrier coherence and Hall effect in organic semiconductors
Source: Sci Rep. 2016 Mar 30;6:23650. doi: 10.1038/srep23650 (PMC4812289; doi:10.1038/srep23650)
Supplement: Supplementary Information [file srep23650-s3.docx]

**Charge carrier coherence and Hall effect in organic semiconductors**

H. T. Yi^1^, Y. N. Gartstein^2^ and V. Podzorov^1,3^

^1^ Dept. of Physics, Rutgers University, Piscataway, NJ 08854, USA; ^2^ Dept. of Physics, University of Texas at Dallas, Richardson, TX, USA. ^3^ Inst. for Adv. Mater. & Devices for Nanotech, Rutgers University, Piscataway, NJ 08854, USA.

^*^ Corresponding author: podzorov@physics.rutgers.edu

**Supplementary Information**

Supplementary file 1 - Origin file with a 3D plot of Eq. (12), describing the degree of discrepancy between the Hall effect and FET measurements as a function of the fraction of band carriers and the ratio of hopping and band mobilities.
 
Supplementary file 2 - Origin file with a 3D plot of Eq. (19), describing the carrier coherence factor as a function of the fraction of band carriers and the ratio of hopping and band mobilities.
